# Etiological factors and clinical outcomes in extracapsular and intracapsular hip fractures among older adults: A gender‐specific analysis

**DOI:** 10.1002/pmrj.13326

**Published:** 2025-02-05

**Authors:** Radcliffe Lisk, Keefai Yeong, David Fluck, Jonathan Robin, Christopher H. Fry, Thang S. Han

**Affiliations:** ^1^ Department of Orthopaedic Trauma Ashford and St Peter's NHS Foundation Trust Chertsey UK; ^2^ Department of Cardiology Ashford and St Peter's NHS Foundation Trust Chertsey UK; ^3^ Department of Acute Medicine Ashford and St Peter's NHS Foundation Trust Chertsey UK; ^4^ School of Physiology, Pharmacology and Neuroscience University of Bristol Bristol UK; ^5^ Department of Endocrinology Ashford and St Peter's NHS Foundation Trust Chertsey UK; ^6^ Institute of Cardiovascular Research, Royal Holloway University of London Egham UK

## Abstract

**Background:**

Compared to patients with intracapsular fractures (ICFs), those with extracapsular fractures (ECFs) had worse outcomes. However, most studies of risk factors for these fractures lacked relevant potential reasons, particularly nutritional status, and adjustment for confounding factors. Furthermore, less is known about their effects on clinical outcomes.

**Objective:**

To conduct a gender‐specific analysis of community‐dwelling individuals admitted with hip fractures to examine the association of clinical risk factors and health care measures.

**Design:**

Monocentric cross‐sectional study.

**Setting:**

Orthopedic trauma department.

**Participants:**

A total of 787 women and 318 men of similar mean age (±SD): 83.1 years (±8.6) and 82.5 years (±9.0), respectively.

**Main Outcome Measures:**

Multivariable logistic regression analyzed risk factors including age, gender, dementia, stroke, ischemic heart disease, diabetes, prefracture mobility, alcohol consumption, American Society of Anesthesiologists grades, drug history, and nutrition status for assessing risk factors and outcomes associated with ECFs and ICFs.

**Results:**

Compared to ICFs, for each additional year of age, women had a 3% and men 4% greater association with ECFs. Among women only, ECFs were associated with risk of malnutrition: odds ratio [OR] = 1.70 (95% CI, 1.17–2.48) or malnourishment: OR = 1.93 (95% CI, 1.06–3.52), stroke: OR = 1.85 (95% CI, 1.16–2.97), and diabetes: OR = 1.92 (95% CI, 1.21–3.06). Women with ECFs were less likely to be discharged to their own homes: OR = 0.56 (95% CI, 0.38–0.83); but more likely to be discharged to a rehabilitation unit: OR = 1.81 (95% CI, 1.21–2.71) and readmitted to hospital within 30 days of discharge ≥1 time: OR: 2.39 (95% CI, 1.27–4.50) or ≥2 times: OR = 3.48 (95% CI, 1.05–11.57): they did not differ in discharge to residential or nursing care or in‐hospital mortality. Among men, there were no differences in discharge destinations or readmissions between types of fractures.

**Conclusions:**

Compared to ICFs, a greater number of risk factors associated with ECFs were identified more often in women than in men, and ECFs also have greater influences on clinical outcomes in women.

## INTRODUCTION

Hip fractures, commonly occurring in older adults as a result of a fall, are one of the leading causes of hospitalization^1^ and are classified as intracapsular or extracapsular. Intracapsular fractures (ICFs) occur within the capsule of the hip joint and extracapsular fractures (ECFs) occur distal to the hip joint capsule. ECFs mostly lie in the intertrochanteric region, with a small proportion in the subtrochanteric region, lying between the lesser trochanter and 5 cm distal to the lesser trochanter.^2^ Most studies indicate that, compared to ICFs, ECFs are associated with worse clinical outcomes, including greater risk of death at 1 year and decreased mobility.^3^ Furthermore, ICFs are associated with a shorter hospital length of stay (LOS)^4^ and a better rate of independent walking at 1 year after hip surgery, whereas the rate of institutionalization is higher for ECFs.^5^ Disparities in outcomes between the two types of fractures led researchers to seek their etiologies, such as age, underlying health conditions, physical functioning,^4^, ^6^ nutritional status,^7^ bone mineral density (BMD),^8^, ^9^ and gender.^10^ Most previous studies were of small sample sizes and contained limited numbers of potential causal factors in their analysis. Furthermore, many studies often combined both genders in their analyses despite evidence indicating that bone structure/composition differs between genders.^11^ Thus, such inadequate adjustment may have led to misleading or contradictory results.

Despite their well‐documented differences, there is a lack of research on the impact of the two types of hip fractures on health care measures relating to discharge planning including: recovery from the hip injury, hospital LOS, discharge destinations, and readmission frequency.^12^ Discharge planning relies largely on the speed of functional recovery from hip injuries (ie, regaining mobility), as well as the availability of the intended discharge destination, which determines hospital LOS. Functional recovery is a crucial indicator for planning the most appropriate placement. Usually, patients who achieve adequate recovery are suitable for return to their own home. Those with a potential to regain their function, but require extra support, are transferred to a rehabilitation unit, whereas those who are unlikely to recover from injury would require long‐term support such as residential or nursing care. Hospital LOS is not only an important health‐quality indicator, but prolonged LOS incurs a huge cost to health care expenditures.^13^

In this study, we conducted an analysis of patients admitted to hospital with hip fractures from their own home to examine the risk factors and clinical outcomes associated with ICFs and ECFs.

## METHODS

### Study design, participants and setting

This cross‐sectional study recruited individuals consecutively admitted to a National Health Service (NHS) hospital with hip fractures (April 2009–June 2019). This study participates in the National Hip Fracture Database (NHFD) audit program, managed by the Royal College of Physicians, which is a quality improvement initiative commissioned by the HealthCare Quality Improvement Partnership, which in turn is commissioned by NHS England. The NHFD data are collected under section 251 of the NHS Act 2016 following approval by the Health Research Authority Confidentiality Advisory Group (CAG 8‐03(PR11)/2013).^14^ Ethical approval was not sought in line with Governance Arrangements for Research Ethics Committee guidance for this secondary analysis of anonymized data.^15^

### Data collection

Data were prospectively collected by a trauma coordinator according to the NHFD protocol,^16^ comprising clinical characteristics including demographic factors, comorbidities, drug history, and nutritional status using Malnutrition Universal Screening Tool (MUST) protocol (Table S1).^17^ Comorbidities including dementia, ischemic heart disease (IHD), stroke, and diabetes were identified from electronic record databases by *International Classification of Diseases, Tenth Revision* disease codes.^18^ Prefracture mobility status was assessed by a standardized tool^19^, ^20^ and physical status by the American Society of Anesthesiologists (ASA) classification.^21^ Outcomes measures were recorded for LOS spent in hospital and in rehabilitation, in‐hospital mortality, recovery (mobility within 1 day of hip surgery in a subsample of 541 patients), discharge destinations, and readmission frequency within 30 days of initial discharge.

### Categorization of variables

The Garden classification was used for describing ICFs (type I–IV), and Association of Osteosynthesis classification for ECFs (trochanteric: A1–A3, and subtrochanteric fractures) (Table S2).^22^ Polypharmacy was defined as ≥4 different types of medications taken daily.^23^ The anticholinergic burden (ACB) score was based on the list of medications developed by the Aging Brain Program.^24^ Antiresorptive agents were categorized into treatment before hip fracture, newly prescribed after hospital admission, and pending for a radiological report. Recommended alcohol consumption was based on government guidelines (<14 units/week).^25^ Categorization of ASA was examined in patients with a grade <3 against those a grade ≥3 (grade 3 indicates severe systemic disease and grade 4 severe systemic disease that is a constant threat to life).^21^ Nutritional status was stratified as well nourished, at risk of malnutrition, and malnourished^17^, ^26^ and readmission as patients who returned to hospital ≥1 or ≥2 times within 30 days of a hospital discharge.

### Statistical analysis

Gender‐specific analysis was performed to compare differences in risk factors between ICF and ECF groups: Mann–Whitney tests for assessing differences in continuous variables, and chi‐square tests for categorical variables. To determine etiological risk factors associated with ECFs compared to ICFs, a *forward procedure* was applied in multivariable logistic stepwise regression by adding each etiological risk factor (independent variable) to the model including age, comorbidities (dementia, stroke, diabetes, IHD, ASA, anticholinergic burden, polypharmacy, alcohol consumption, nutritional status, and prefracture mobility). Each was added separately and retained if their inclusion created the most statistically significant improvement of the fit to the data, and the process was repeated, adding on to the model, until no further statistically significant improvements occurred. This produced a model that included all of the variables that are statistically significantly related to the outcome.

To determine the likelihood of a discharge back home or rehabilitation or readmissions (outcomes) in women and in men with ECFs compared to those with ICFs, two models of logistic regression using *enter procedure* were conducted. Model 1: unadjusted, and model 2: adjusted for potential confounding factors (etiological risk factors listed previously), all of which were forced into the model. Results were expressed in odds ratio (OR) and 95% confidence interval (CI). The statistical significance threshold was accepted as *p* < .05. Statistical analyses were performed using SPSS Statistics for Windows, Version 28.0 (IBM Corp, Armonk, NY).

## RESULTS

Table 1 shows characteristics of all 1105 patients; 787 (71.2%) women and 318 (28.8%) men of similar mean ages, 83.1 years (SD = 8.6) and 82.5 years (SD = 9.0). There were only marginally (nonsignificant) higher proportions of women with a history of dementia, prefracture limited mobility, and treatment with an antiresorptive agent before admission, whereas there were significantly higher proportions of men with a history of IHD (*p* = .002), stroke (*p* = .001), alcohol consumption >14 units/week (*p* = .001), and polypharmacy (*p* = .018). There were higher proportions of women discharged to residential/nursing care (*p* = .019), whereas higher proportions of men died in hospital (*p* = .009) and or were readmitted within 30 days of hospital discharge (*p* = .011).

**TABLE 1:** Characteristics of patients admitted with hip fractures.

	All (*n* = 1105)		Women (*n* = 787)		Men (*n* = 318)		*χ* ^2^ test^b^
	*n*	%	*n*	%	*n*	%	*p* value
Age (years)							
60–69.9	108	9.8	77	9.8	31	9.7	.753
70–79.9	251	22.7	175	22.2	76	23.9	
80–89.9	510	46.2	371	47.1	139	43.7	
≥90	236	21.4	164	20.8	72	22.6	
Intracapsular fractures (all)	640	57.9	459	58.3	181	56.9	.359
Undisplaced (types I and II)	584	91.3	43	9.4	13	7.2	.151
Displaced (types III and IV)	56	8.7	416	90.6	168	92.8	
Extracapsular fractures (all)	465	42.1	328	41.7	137	43.1	.359
Intertrochanteric – grade A1/A2	362	77.8	256	78.0	106	77.4	.860
Intertrochanteric – grade A3 (reverse oblique)	31	6.7	21	6.4	10	7.3	
Intertrochanteric – other	33	7.1	25	7.6	8	5.8	
Subtrochanteric	39	8.4	26	7.9	13	9.5	
Morbidities and medications							
Dementia	223	20.2	167	21.2	56	17.6	.101
Ischemic heart disease	114	10.3	67	8.5	47	14.8	.002
Stroke	157	14.2	95	12.1	62	19.5	.001
Diabetes	143	12.9	97	12.3	46	14.5	.194
Alcohol consumption >14 units a week	58	5.3	30	3.8	28	8.8	.001
ASA score ≥ 3	660	62.4	461	58.6	199	62.6	.001
ACB score ≥ 1	429	38.8	304	38.6	125	39.3	.443
Polypharmacy (≥4 daily medications)	827	74.8	575	73.1	252	79.2	.018
Antiresorptive agents before admission	45	4.1	36	4.6	9	2.8	.201
Antiresorptive agents newly prescribed on admission	896	81.1	641	81.8	255	80.4	
Antiresorptive agents waiting for radiological report	160	14.5	107	13.6	53	16.7	
Prefracture mobility status							
Freely mobile without aids	502	45.4	356	45.2	146	45.9	.159
Mobile outdoors with one aid	254	23.0	179	22.7	75	23.6	
Mobile outdoors with two aids or frame	121	11.0	84	10.7	37	11.6	
Some indoor mobility but never goes outside without help	223	20.2	164	20.8	59	18.6	
No functional mobility of lower limbs	2	0.2	2	0.3	0	0	
Nutritional status							
Well‐nourished	731	66.2	509	64.7	222	69.8	.159
Risk of malnutrition	204	18.5	156	19.8	48	15.1	
Malnourished	72	6.5	52	6.6	20	6.3	
Recovery							
Failure to mobilize within 1 d of surgery^a^	191	35.3	126	33.2	65	40.4	.066
Discharge destinations							
Died in hospital	52	4.7	29	3.7	23	7.7	.009
Back to own home	581	52.6	422	54.4	159	51.6	.225
Rehabilitation units	360	32.6	250	32.2	110	35.7	.151
Residential or nursing care	61	5.5	51	6.6	10	3.2	.019
Others	51	4.6	24	3.1	6	1.9	.206
Readmission within 30 d of hospital discharge							
≥1 readmission	117	10.6	72	9.1	45	14.2	.011
≥2 readmission	29	2.6	18	2.3	11	3.5	.184
Length of stay (days)	Median	IQR					
In hospital	10.2	6.6–16.1	9.7	6.7–15.2	11.3	6.5–18	.066^c^
In rehabilitation	24	15–37	24	16–36.5	23	13.8–38	.871^c^

Abbreviations: ACB, anticholinergic burden; ASA, American Society of Anesthesiologists.

^a^ Subsample of 541 patients.

^b^ Chi‐square test.

^c^ Mann–Whitney *U* test for comparing characteristic differences between men and women.

**FIGURE 1:**
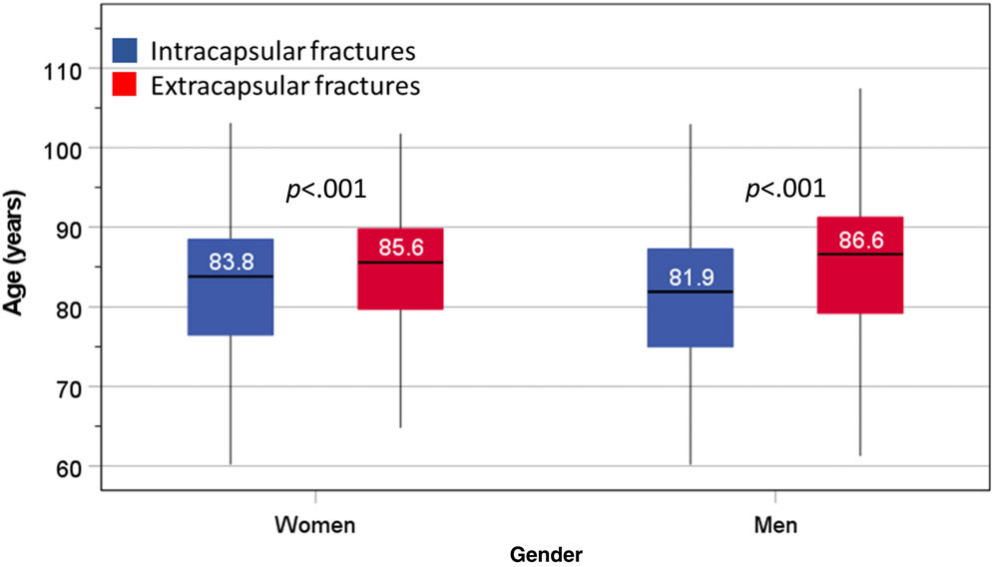
Box and whisker plot of age in men and women according to types of fractures. Bar representing median, box representing interquartile ranges and whiskers representing 5th and 95th percentiles.

Patients with ICFs had a significantly lower median (interquartile range) age than those with ECFs in women: 83.8 years (78–89) versus 85.6 years (80–90) and in men: 81.9 years (75–87) versus 86.6 years (79–92) (Figure 1). Among women, compared to those with ICFs, higher proportions of women with ECFs had a previous stroke (10.2% vs. 14.6%, *p* = .040), diabetes (10.5% vs. 14.9%, *p* = .038), prefracture mobility limited to indoors (28.8% vs. 36.1%, *p* = .019), and polypharmacy (69.7% vs. 77.7%, *p* = .007) and were prescribed with an antiresorptive agent before hip fracture (3.1% vs. 6.7%, *p* = .012) and a risk of malnutrition (18.2% vs. 27.1%) or malnourished (5.8% vs. 9.4%, *p* < .001). In men, other than an association with age (discussed later), there were no differences in either type of hip fractures for all risk factors analyzed, including comorbidities, drug history, mobility limitations, anesthetic risk, or nutritional status (Table 2).

**TABLE 2:** Comparison between intracapsular and extracapsular fractures in 1105 patients admitted with hip fractures.

	Women (*n* = 787)				Men (*n* = 318)			
	Intracapsular fractures (58.3%)	Extracapsular fractures (41.7%)	*χ* ^2^	*p* value	Intracapsular fractures (56.9%)	Extracapsular fractures (43.1%)	*χ* ^2^	*p* value
Morbidities								
Dementia	20.7	22.0	0.2	.368	17.7	17.5	0.1	.546
Ischemic heart disease	7.4	10.1	1.7	.118	16.0	13.1	0.5	.290
Stroke	10.2	14.6	3.5	.040	22.1	16.1	1.8	.114
Diabetes	10.5	14.9	3.6	.038	15.5	13.1	0.3	.337
Alcohol consumption >14 units/week	3.7	4.0	0.1	.496	11.0	5.8	2.6	.076
Prefracture mobility limited to indoors	28.8	36.1	4.7	.019	27.8	33.6	1.2	.161
ASA score ≥ 3	60.0	63.9	1.2	.157	62.2	64.2	0.1	.545
ACB score ≥ 1	36.4	41.8	2.3	.073	35.9	43.8	2.0	.095
Polypharmacy (≥4 daily medications)	69.7	77.7	6.3	.007	81.2	76.6	1.0	.196
Antiresorptive agents before admission	3.1	6.7	5.9	.012	1.7	4.4	2.1	.136
Nutritional status								
Risk of malnutrition	18.2	27.1	13.0	.001	17.5	15.3	1.4	.487
Malnourished	5.8	9.4			5.4	8.9		
Recovery								
Failure to mobilize within 1 d of surgery	29.4	38.9	3.7	.055	44.1	35.3	1.3	.168
Discharge destinations								
Died in hospital	3.5	4.0	0.1	.437	9.8	4.5	3.1	.060
Back to own home	61.5	44.4	22.1	<.001	50.6	53.0	0.2	.380
Rehabilitation	26.3	40.4	17.2	<.001	34.5	37.3	0.3	.346
Residential/nursing care	6.0	7.4	0.6	.257	3.4	3.0	0.1	.544
Readmissions								
≥1 readmission within 30 d of discharge	5.4	14.3	18.2	<.001	15.5	12.4	0.6	.271
≥2 readmission within 30 d of discharge	1.1	4.0	7.1	.008	3.3	3.6	0.0	.553

Abbreviations: ACB, anticholinergic burden; ASA, American Society of Anesthesiologists.

**FIGURE 2:**
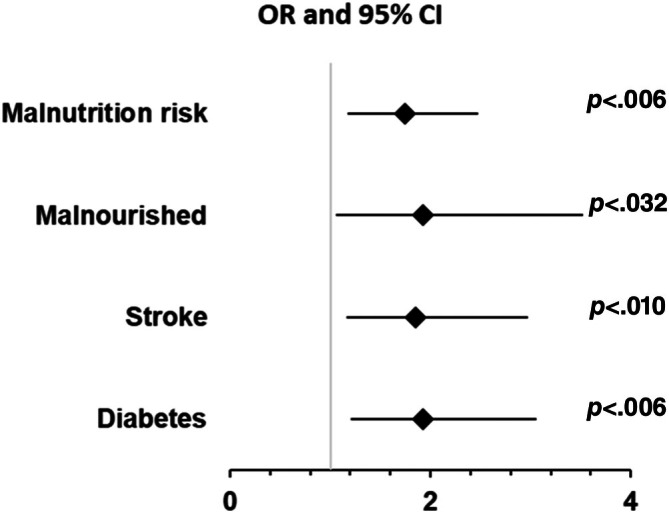
Multivariable stepwise logistic regression analysis using *forward* selection procedure to determine relevant risk factors of extracapsular fractures in women. CI, confidence interval; OR, odds ratio.

Multivariable forward stepwise logistic regression showed that for each additional year of age, women had a 3% (OR = 1.03 [95% CI, 1.01–1.05]) and men a 4% (OR = 1.04 [95% CI, 1.01–1.07]) greater association with ECFs compared to ICFs. In women only, ECF was also associated with those at risk of malnutrition: OR = 1.70 (95% CI, 1.17–2.48) or being malnourished: OR = 1.93 (95% CI, 1.06–3.52) or having stroke: OR = 1.85 (95% CI, 1.16–2.97) and diabetes: OR = 1.92 (95% CI, 1.21–3.06) (Figure 2).

### Types of hip fractures and mortality, recovery, and LOS

**FIGURE 3:**
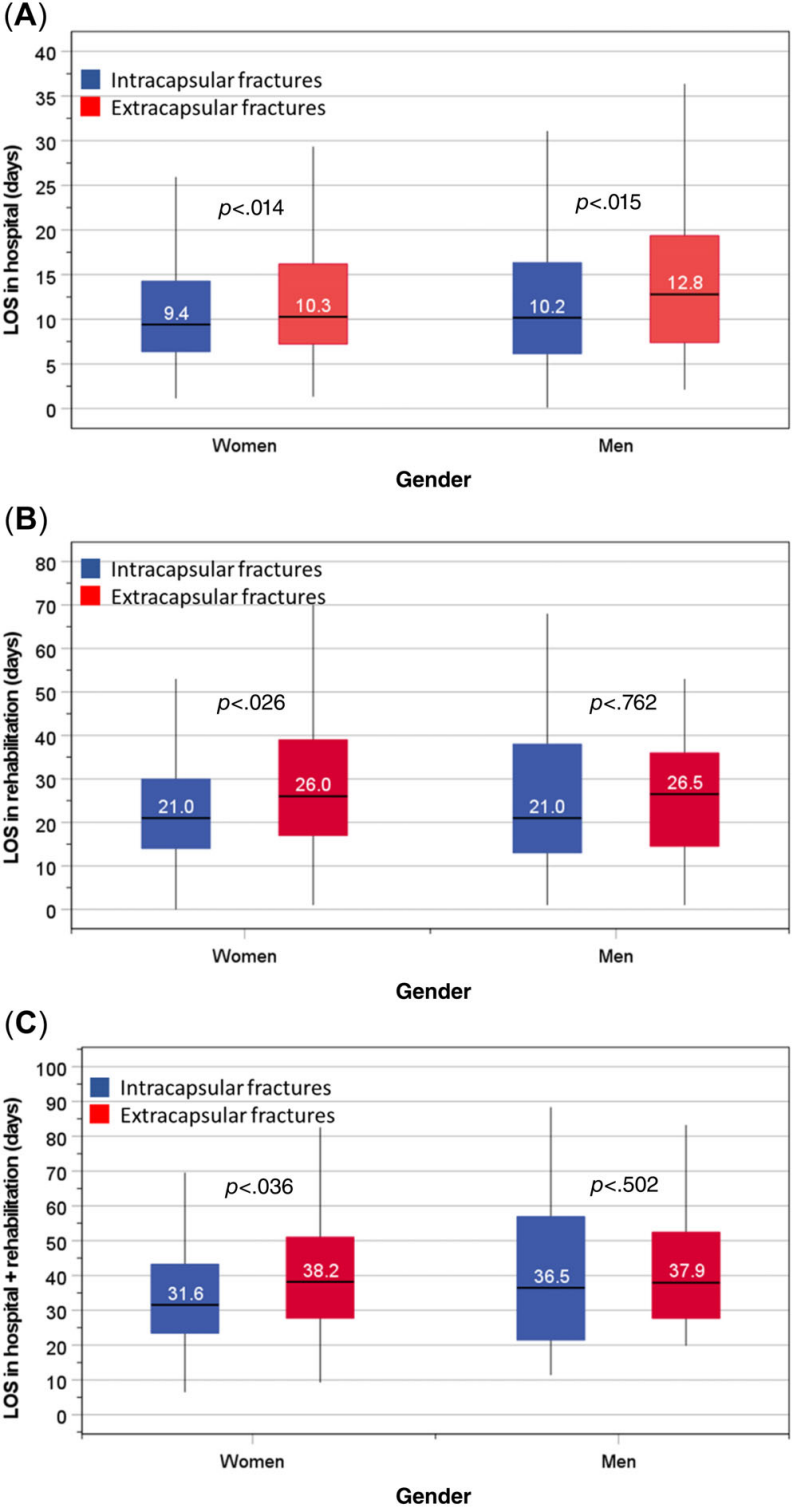
Comparison of length of stay in hospital (A), in rehabilitation (B), and both in hospital and rehabilitation (C) between patients with intracapsular fractures and those with extracapsular fractures. LOS, length of stay.

For either gender, rates of in‐hospital mortality or recovery after hip surgery did not differ between types of hip fracture. Compared to patients with ICFs, those with ECFs had significantly longer median LOS in hospital: women; 10.3 days versus 9.4 days (*p* = .014) and men; 12.8 days versus 10.2 days (*p* = .015) (Figure 3A). The median LOS in rehabilitation was also significantly longer for women with ECFs: 26.0 days versus 21.6 days (*p =* .026), but this difference did not achieve significance in men: 26.5 days versus 21.0 day (*p* = .762) (Figure 3B), which may be due to smaller numbers in men, thus wider interquartile range. Similarly, the median LOS both in hospital and rehabilitation was longer only for women with ECFs: 38.2 versus 31.6 days (*p* = .036) (Figure 3C).

### Types of hip fractures and discharge destinations and readmission

Compared to women with ICFs, women with ECFs were less likely to be discharged to their own homes: rates = 42.1% versus 61.8%, OR = 0.56 (95% CI, 0.38–0.83) but were more likely to be discharged to a rehabilitation unit: 43.2% versus 26.3%, OR = 1.81 (95% CI, 1.21–2.71). They were also more likely to be readmitted to hospital within 30 days of discharge ≥1 time: 43.2% versus 26.3%, OR = 2.39 (95% CI, 1.27–4.50) or ≥2 times: 7.1% versus 4.0%, OR = 3.48 (95% CI, 1.05–11.57). However, discharge to residential/nursing care did not differ between types of hip fractures. There were no differences in discharge destinations or readmissions between different types of fractures in men (Tables 2 and 3).

**TABLE 3:** Logistic regression analysis using enter method to determine the likelihood of a discharge back home or rehabilitation, or readmissions in women and in men with extracapsular fractures compared to those with intracapsular fractures.

	OR	95% CI	*P* value
Women			
Discharge destinations (unadjusted)			
Back to own home	0.50	0.38–0.67	<.001
Rehabilitation	1.90	1.40–2.58	<.001
≥1 readmission within 30 d of discharge	2.90	1.75–4.83	<.001
≥2 readmission within 30 d of discharge	3.75	1.32–10.82	.013
Discharge destinations (adjusted)^a^			
Back to own home	0.56	0.38–0.83	.004
Rehabilitation	1.81	1.21–2.71	.004
≥1 readmission within 30 d of discharge	2.39	1.27–4.50	.007
≥2 readmission within 30 d of discharge	3.48	1.05–11.57	.042
Men			
Discharge destinations (unadjusted)			
Back to own home	1.10	0.70–1.73	.675
Rehabilitation	1.13	0.71–1.81	.607
≥1 readmission within 30 d of discharge	0.77	0.41–1.48	.439
≥2 readmission within 30 d of discharge	1.11	0.33–3.79	.872
Discharge destinations (adjusted)^a^			
Back to own home	1.21	0.65–1.24	.547
Rehabilitation	1.41	0.74–2.67	.299
≥1 readmission within 30 d of discharge	0.63	0.26–1.57	.516
≥2 readmission within 30 d of discharge	0.86	0.18–4.17	.853

Abbreviations: CI, confidence interval; OR, odds ratio.

^a^ Adjusted for age, comorbidities (dementia, stroke, diabetes, ischemic heart disease), American Society of Anesthesiologists (grade ≥ 3), anticholinergic burden (score ≥ 1), polypharmacy (≥4 daily drugs), alcohol consumption (>14 units a week), nutritional status, and prefracture mobility limitations to indoors. Data for mortality and discharge to residential/nursing care not shown (no association with type of fractures in either men or women).

## DISCUSSION

### Summary of findings

In this gender‐stratified study, age, risk of malnutrition and malnourishment, stroke, and diabetes were the principal risk factors for ECFs in women. Alternatively, age was the only risk factor for ECFs in men. Compared to those admitted with an ICF, women with an ECF were less likely to be discharged back to their own home, more likely to need rehabilitation, stayed longer in hospital and rehabilitation, and were more likely to be readmitted to hospital within 30 days of a discharge. In men, except for an association with longer LOS in hospital with ECFs, there were no other differences in outcome measures between ICFs and ECFs. Furthermore, our data agree with existing evidence to show that aging, chronic medical conditions, and malnutrition have greater influences on having an ECF than an ICF. With advancing age individuals tend to accumulate more chronic health conditions that are inextricably linked. As far as we are aware, this study included a more complete dataset of risk factors, which enabled us to conduct multivariable stepwise logistic regression to select the most relevant independent risk factors. This technique, in conjunction with stratification for gender, minimizes bias and misleading results that follow with the inclusion of only a limited number of variables.

### Gender, age, and incidence of hip fractures

The gender distribution (71.2% women; 28.8% men) was similar to that from UK national data (72.7% women; 27.3% men).^27^ Women and men had similar higher rates of ICFs than ECFs, but etiological risk factors differed. Multivariable logistic analysis showed there were more risk factors associated with ECF in women (malnutrition risk/malnourishment, stroke, and diabetes) than in men. This is important as it highlights that comorbidities should receive greater attention in women to prevent more severe ECFs. The reasons for an age difference between ICFs and ECFs remain uncertain and other unaccounted risk factors may be present.

### Hip fractures; nutritional factors, bone mineral density, and polypharmacy

Malnutrition in older adults is associated with osteoporosis, low BMD, and hip fractures,^28^ but little is known about the effect of malnutrition on the type of fracture. However, BMD is lower in patients with ECF than for those with ICFs^8^, ^9^; in particular low femoral neck BMD is associated with an increased risk of ECFs.^29^ These findings support an association between ECFs and patients with a malnutrition risk or malnourishment, particularly in women, as observed in this study. However, counterobservations by other groups showed that malnutrition, as indicated by a low body mass index, was more common in patients with ICFs^7^ or that the proportion of patients with ECFs showed no dependency on the level of nourishment.^30^

Polypharmacy and antiresorptive agents were associated with higher prevalences (unadjusted) of ECFs compared to ICFs in women. However, these two factors were not selected as risk factors by stepwise regression analysis for this study. Polypharmacy reflects underlying poor health and was not used as an independent variable. The relationship between antiresorptive treatment and osteoporosis is more complex. Patients diagnosed with osteoporosis may receive an antiresorptive agent, but many patients are not diagnosed. In this study only 4.1% of patients were being treated with an antiresorptive agent upon admission, whereas most (81.1%) were newly prescribed on discharge; the remainder awaited radiological reports. The higher proportion of patients with ECFs prescribed with an antiresorptive agent was unlikely to be causally linked, an association also seen in a large U.S. study^29^ and is more likely due to prescription of patients with a history of osteoporosis or recurrent bone fractures.

It would be of interest to include BMD and osteoporosis in our analysis. However, a large proportion of patients did not have BMD assessed prior to admission, and for many dual‐energy X‐ray absorptiometry (DXA) was arranged for postdischarge, reflecting a wider inadequacy of BMD monitoring for at‐risk individuals. In a study of just over 500 patients admitted with hip fractures to four U.S. health centers, only 12%–24% had BMD assessed by DXA and 7%–37% received an antiresorptive agent after hip fractures.^31^ In another study of 268 adults in Germany, DXA scan within 3 months of arthroplasty revealed 49 patients (18%) had osteoporosis, of whom 36 (73%) were not previously diagnosed with osteoporosis.^32^ These findings highlight the need for improved BMD monitoring for at‐risk individuals, such as those with underlying chronic conditions or malnutrition.

### Stroke effects on hip fractures

A previous stroke is a risk factor for hip fractures,^33^, ^34^ possibly with motor, sensory, and visual impairments as contributing factors, and our study extended this by showing specifically a greater risk for ECFs. Information on the hemiparetic side of the stroke was unavailable; therefore, the association between the affected limb from stroke and hip fracture could not be assessed. The reason for the increased risk of patients with an ECF may in part be due to underuse of the lower limb (particularly on the paretic side) after a stroke, resulting in sarcopenia and bone loss. These factors predispose such patients to falls and thus fractures are more common on the paretic side.^34^ In patients with a hemiplegic stroke, low BMD is preferentially associated with ECFs on the paretic side.^34^, ^35^

### Hip fractures and diabetes

This study showed a greater association between ECFs than ICFs and those with a history of diabetes and complements other reports of a high risk of bone fractures in patients with type 1 or type 2 diabetes. We also observed that high proportions (>93%) of patients with diabetes or stroke were prescribed ≥4 daily drugs, with corresponding proportions of an ACB score ≥1 of 46.2% and 46.5%. These factors are compounded by changes to bone loss in type 1 diabetes and bone fragility in type 2 diabetes.^36^, ^37^ In addition, the use of thiazolidinediones to treat type 2 diabetes is associated with an increased risk of bone fractures.^36^ Our data showed diabetes had a significant effect on ECFs in women, but not in men, and suggest that diabetes contributes equally to both types of fractures in men. However, these findings do not rule out diabetes as a risk factor for osteoporosis or overall hip fractures in men.

### Health care and financial outcomes of hip fractures

Previous studies on the association between types of hip fractures and clinical outcomes focused primarily on functional recovery and postfracture mortality. By contrast, there is a lack of data on discharge planning and its influences on care delivery, hospital capacity, and expenditure.^38^ Several factors determine discharge planning and hospital LOS. The finding that a higher proportion of women with ECFs were discharged to rehabilitation is poorly documented, but the fact that fewer patients returned to their own home concurs with a previous study.^3^ We found no differences in functional recovery and in‐hospital mortality between ECFs and ICFs. This suggests that hospital LOS may, in part, be due to a wait for residential placement. Our findings agree with a previous study that patients with ECFs had a longer LOS in orthopedic wards and in hospital overall.^4^ Prolonged LOS in hospital can be detrimental to patients, for example, due to loss in muscle strength^39^ and increased risk of nosocomial infections,^40^ with a significant impact on health care costs. By contrast, timely discharge is associated with better patient outcomes.^41^

In this study, almost a third of patients required rehabilitation, and women with ECFs spent significantly longer in rehabilitation units. These findings have not been previously reported and are important with respect to additional health care services expenditure.^38^ Furthermore, readmission rates for women with ECFs were greater. A large 2020 study estimated additional annual secondary care costs for patients with hip fracture, compared to those admitted for other reasons, were about £635 m.^42^ In addition, annual rehabilitation costs were estimated in 2017 at about £12 k.^43^ There are no estimates for different types of hip fractures but given their longer LOS both in hospital and in rehabilitation, a patient with ECF, compared to a patient with ICF, will incur higher unit costs to the NHS.

The reasons for worse outcomes among patients with ECF are multifactorial. However, they are generally older and so more often have coexisting health conditions such as stroke,^34^ diabetes,^44^ and malnutrition risk/malnourishment. ECFs may also reflect a more severe injury, for example, from a heavy fall. Different underlying factors may also determine the two types of hip fractures; ICFs may be more pathophysiologically related, such as poor vascularization of the femoral head or a lack of periosteum, whereas ECFs are more related the mechanical properties of bone.^12^ The worse outcomes in women indicate potential differences in underlying etiologies between males and females. Low BMD is more strongly associated with ECFs,^45^ and osteoporosis occurs more commonly in older women.^46^ In addition, a longer LOS in women may be due, in part, to societal factors, such as less spousal support on returning home after a hip fracture due to their longer life expectancy^47^ and the fact that more men than women find a new partner after a spouse's death.^48^, ^49^ Despite an adjustment for potential confounding factors, the adverse association of ECF with outcomes remained significant suggesting other unaccounted factors. Our study offers further evidence of the need to reduce ECFs, particularly in women.

### Strengths and limitations

The strengths of this study lie in its relatively large number of patients whose data were collected using standardized protocols, including the MUST protocol for assessing nutritional status.^14^, ^16^ To reduce bias from individuals living in residential or nursing care who were likely to have poorer health, only patients admitted from their own homes were selected, although information on independent living was lacking. The analysis was adjusted for confounding factors including chronic conditions such as dementia, stroke, IHD, and diabetes as well as functional limitations prior to a hip fracture. Information on ethnicities is not routinely collected by the NHFD audit program, but a study of stroke survivors admitted to our hospital over a similar period showed 5.6% were from ethnic minorities.^50^ In addition, information on smoking was not available. Caution should also be taken when extrapolating data from this study to the general population as data were from a single center.

In conclusion, our findings indicate a need for reducing and preventing hip fractures, with a greater emphasis on identification and managing risk factors, including higher levels of support for older adults, especially in women, in those with comorbidities such as stroke and diabetes, as well as those at risk of malnutrition.

## DISCLOSURES

The authors declare that they have no conflict of interest.

## PATIENT CONSENT STATEMENT

Informed consent was obtained from all individual participants included in the study.

## Supporting information


**Data S1.** Supporting Information.
