# Supplementary material for: Etiological factors and clinical outcomes in extracapsular and intracapsular hip fractures among older adults: A gender‐specific analysis
Source: PM R. 2025 Feb 5;17(7):793–803. doi: 10.1002/pmrj.13326 (PMC12238746; doi:10.1002/pmrj.13326)
Supplement: Supplementary file 1 — Data S1. Supporting Information. [file PMRJ-17-793-s001.doc]

**Supplementary Table 1.** Classification of nutrition status adopted from MUST screening tool.17

| **Malnutrition Universal Screening Tool** | | | | | |
| --- | --- | --- | --- | --- | --- |
| **Phenotypic criteria** | | | | **Etiologic criteria** | |
| **Body mass index (kg/m^2^)** | **Score** | **Unintentional weight loss (%)** | **Score** |  | **Score** |
| >20 (>30) | 0 | <5 | 0 | If patient is acutely ill and there has been or is likely to be no nutritional intake for >5 days | 2 |
| 18.5-20 | 1 | 5-10 | 1 |
| <18.5 | 2 | >10 | 2 |

Overall score = 0 indicates low risk of malnutrition. Overall score = 1 indicates medium risk of malnutrition. Overall score of ≥2 indicates high risk of malnutrition.

**Supplementary Table 2.** Classification of hip fractures.22

| **Intracapsular fracture (Garden’s classification)** | **Extracapsular fracture** |
| --- | --- |
| - Type I: incomplete fracture and no displacement | - Trochanteric fractures (AO classification) |
| - Type II: complete fracture and no displacement | - A1: two-part, stable fracture |
| - Type III: complete fracture and partial displacement | - A2: comminuted, unstable fracture |
| - Type IV: complete fracture and complete displacement | - A3: reverse or transverse, unstable fracture |
|  | - Subtrochanteric fractures |
